# The Epidermal Growth Factor Receptor Is Involved in Angiotensin II But Not Aldosterone/Salt-Induced Cardiac Remodelling

**DOI:** 10.1371/journal.pone.0030156

**Published:** 2012-01-23

**Authors:** Smail Messaoudi, An Di Zhang, Violaine Griol-Charhbili, Brigitte Escoubet, Junichi Sadoshima, Nicolette Farman, Frederic Jaisser

**Affiliations:** 1 INSERM, U872, Centre de Recherche des Cordeliers, Paris, France; 2 Pierre et Marie Curie University, Paris VI, Paris, France; 3 Assistance Publique-Hôpitaux de Paris, Hôpital Bichat, Paris, France; 4 University Denis Diderot, Paris 7, Paris, France; 5 Department of Cell Biology and Molecular Medicine, Cardiovascular Research Institute, University of Medicine and Dentistry of New Jersey, New Jersey Medical School, Newark, New Jersey, United States of America; University of Cordoba, Spain

## Abstract

Experimental and clinical studies have shown that aldosterone/mineralocorticoid receptor (MR) activation has deleterious effects in the cardiovascular system; however, the signalling pathways involved in the pathophysiological effects of aldosterone/MR *in vivo* are not fully understood. Several *in vitro* studies suggest that Epidermal Growth Factor Receptor (EGFR) plays a role in the cardiovascular effects of aldosterone. This hypothesis remains to be demonstrated *in vivo*. To investigate this question, we analyzed the molecular and functional consequences of aldosterone exposure in a transgenic mouse model with constitutive cardiomyocyte-specific overexpression of a mutant EGFR acting as a dominant negative protein (DN-EGFR). As previously reported, Angiotensin II-mediated cardiac remodelling was prevented in DN-EGFR mice. However, when chronic MR activation was induced by aldosterone-salt-uninephrectomy, cardiac hypertrophy was similar between control littermates and DN-EGFR. In the same way, mRNA expression of markers of cardiac remodelling such as ANF, BNF or β-Myosin Heavy Chain as well as Collagen 1a and 3a was similarly induced in DN-EGFR mice and their CT littermates. Our findings confirm the role of EGFR in AngII mediated cardiac hypertrophy, and highlight that EGFR is not involved *in vivo* in the damaging effects of aldosterone on cardiac function and remodelling.

## Introduction

Mineralocorticoid excess and MR implication in cardiac diseases have extensively been studied the last decade, and the involvement of MR activation in the development of several heart diseases has been demonstrated through the use of specific MR antagonists (spironolactone, eplerenone) in clinical and experimental studies. The RALES, EPHESUS and EMPHASIS studies demonstrated that MR blockade reduces remarkably mortality in patients with heart failure [1] [2] [3]. Pharmacological MR antagonism also prevents cardiac damages in several animal models such as spontaneously hypertensive rats, uninephrectomized rats treated with aldosterone-salt, heart failure after myocardial infarction or aortic banding [4]. Although progresses have been accomplished, the mechanisms underlying aldosterone-induced contributions to myocardial remodelling and the progression of cardiovascular diseases are still under investigation.

The activation of Epidermal Growth Factor Receptor (EGFR) has been proposed to play a role in the cardiovascular effects of aldosterone, particularly in the crosstalk between aldosterone and Angiotensin II (AngII) [5]. Several studies support a relation between aldosterone and EGFR. *Ex vivo*, aldosterone increased renal, endothelial and vascular smooth muscle cells EGFR expression and/or activation. Furthermore, in these cell types downstream EGFR signaling cascades are induced upon addition of aldosterone or prevented by MR blockade [6]. *In vivo* cardiac and aortic EGFR expressions are increased in adrenalectomized rats treated with aldosterone [7]. We recently demonstrated that EGFR mediates the vascular dysfunction but not the remodelling in uninephrectomized mice treated with aldosterone/salt [8]. However, the role of EFGR activation in the pathogenic effects of aldosterone in the heart *in vivo* remains to be demonstrated.

We hypothesized that EGFR activation might contribute to the damaging effects of aldosterone in the heart *in vivo*. To investigate this question, we analyzed the molecular and functional consequences of aldosterone exposure in a transgenic mouse model with constitutive, cardiomyocyte-specific overexpression of a truncated EGFR protein acting as a dominant negative (DN-EGFR). This model has been successfully used to block cardiac EGFR transactivation by AngII [9].

This mouse model was challenged with either AngII or aldosterone (associated to uninephrectomy and high salt diet). The results obtained in this study confirm the role of EGFR in AngII mediated cardiac hypertrophy, and highlight that EGFR is not involved *in vivo* in the damaging effects of aldosterone/salt on cardiac function and remodelling.

## Methods

### Mice

Mice expressing a dominant negative isoform of EGFR (DN-EGFR) in the cardiomyocytes only were generated on a FVB background, using the α-myosin heavy chain promoter to achieve cardiac-specific expression (see [9] for further details).

Experiments were conducted in accordance with the standard ethical guidelines (National Institutes of Health's “Guide for the care and use of Laboratory animals”, and the European Communities Council European Communities Directive 86/609 EEC. Approval ID: Ce5/2009/034, delivered the 11/12/2009. All experiments involving mice were approved by the Ile de France Regional Ethics Committee for Animal Experiments.

### Angiotensin II treatment

Adult male DN-EGFR mice and wild-type littermates used as controls (CT) were treated with AngII (200 ng/kg/min) or vehicle (0.9% sodium chloride) infused during 2 weeks using osmotic mini-pumps (model 2002, Alzet, Charles River Laboratories, Inc.).

### Nephrectomy aldosterone-salt treatment (NAS)

Adult male DN-EGFR mice and their wild-type littermates (CT) were used. To generate the Aldosterone-salt hypertensive mice, mice underwent left uninephrectomy and were implanted with osmotic minipumps filled with aldosterone (60 µg/kg/d) or vehicle (EtOh10%), (model 2006, Alzet, Charles River Laboratories, Inc.). After one day recovery, animals treated with aldosterone were given 1% NaCl in the drinking water for 2 or 4 weeks.

### Blood pressure, echocardiographic analysis

Systolic BP (SBP) was measured by tail cuff plethysmography in trained conscious mice using a BP2000 Visitech model (Bioseb, Chaville, France). Echocardiography was performed on lightly anesthetized mice (isofluorane, Abott, in oxygen), as previously described [10].

### Organ weights

At sacrifice, heart and kidneys was removed and weighed. Tibia length was measured. Organs were snap frozen in liquid nitrogen and stored at −80°C for molecular analysis.

### Fibrosis

Cardiac cryostat sections (7 µm) were stained with the collagen specific Sirius red stain (0.5% in saturated picric acid). Sections were double-blinded observed in microscope and quantified.

### Quantitative polymerase chain reaction in real time (real time Q-PCR)

Frozen hearts were crushed in Trizol (Invitrogen, Cergy Pontoise, France) in tubes specific for lysis (Lysing D matrix, QBiogene, Illkirch, France). Total RNAs were extracted using phenol chloroform and treated with DNase I (Ambion, Applied Biosystems, Courtaboeuf, France). Reverse-transcription was performed using the reverse transcriptase Superscript II (200 U/µl, Invitrogen, Cergy Pontoise, France) as previously described [10].

Real time Q-PCR was carried out on an iCycler (Biorad Laboratories, Marnes La Coquette, France) using gene-specific primers to quantify the relative abundance of each gene with SYBR Green I as the fluorescent molecule as described [10]. The primers used are listed in Table 1. The ubiquitin (UBC) gene was used as the reference gene for normalization. The relative expression of the target genes was calculated with the 2^(−ΔΔCt)^ method as described [10].

**Table 1:** Primers sequences.

Genes	Forward Primer	Reverse Primer
18S	CGCCGCTAGAGGTGAAATTC	TCTTGGCAAATGCTTTCGC
ANF	CTCGTCTTGGCCTTTTGG	TCGGGGAGGGAGCTAAGT
BNF	CGTCAGTCGTTTGGGCTGTA	GCAGCCAGGCGGTCTTCCT
α-MHC	GCCAAGACTGTCCGGAATGA	TGGAAGATCACCCGGGACTT
β-MHC	CAAAGGCAAGGCAAAGAAAG	TCACCCCTGGAGACTTTGTC
Col1A	CCCCGGGACTCCTGGACTT	GCTCCGACACGCCCTCTCTC
Col3A	CCTGGAGCCCCTGGACTAATAG	GCCCATTTGCACCAGGTTCT
Mmp9	CTGGAAGATGTCGTGTGAGT	CAGGAGTCTGGATAAGTTGG

### Statistics

Values are means ± SEM. Differences between groups were assessed with ANOVA-2 or KRUSKALL-WALLIS tests (Statview software). Values of *p*≤0.05 were considered significant.

## Results

### DN-EGFR mice and AngII treatment

**Figure 1:**
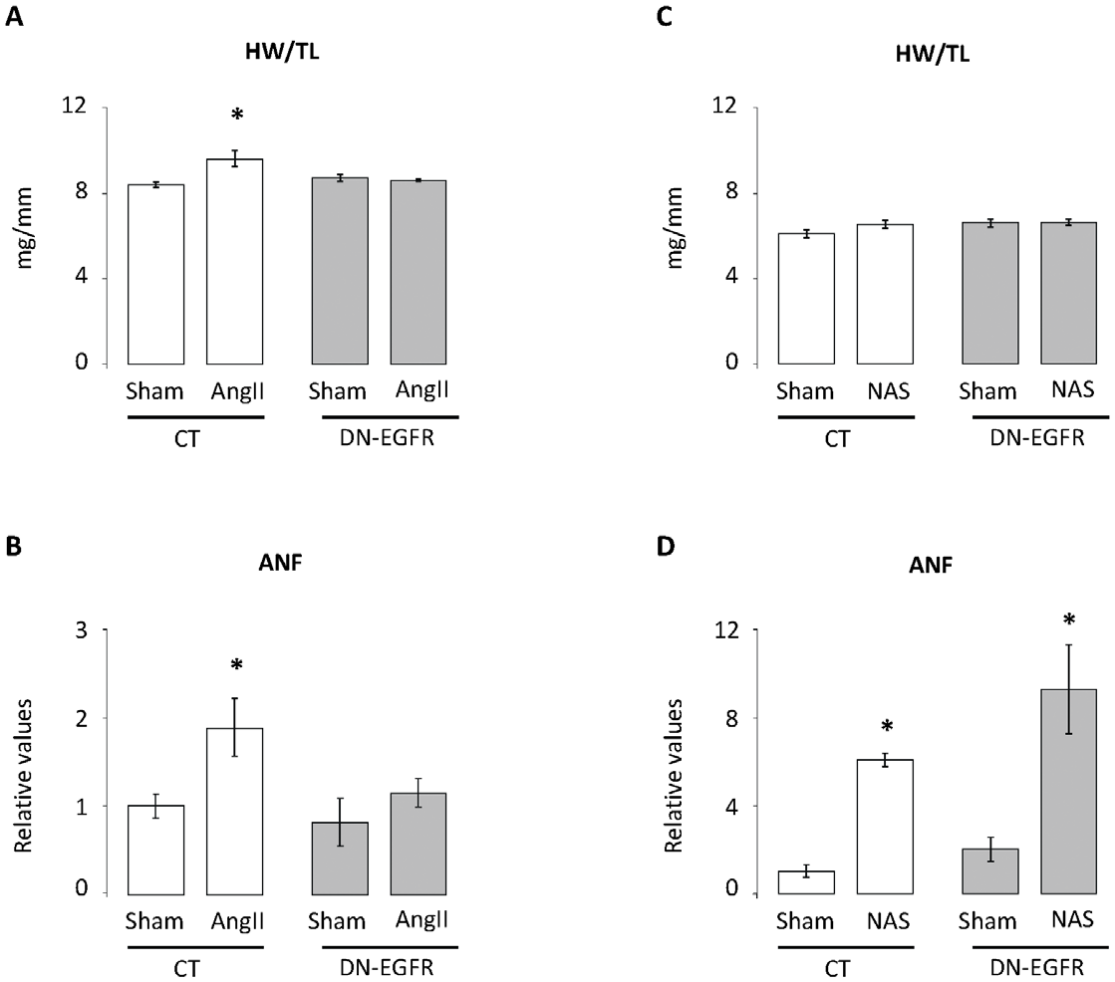
Cardiac phenotype of CT and DN-EGFR mice after two weeks of Angiotensin II or aldosterone (NAS) treatment. **A:** Heart weight to tibia length ratio of CT and DN-EGFR mice under basal conditions or after two weeks of Angiotensin II infusion. Values are means ± SEM, n = 3–6 mice per group. *p<0.05, Angiotensin II-treated mice *versus* corresponding Sham. **B:** Cardiac ANF mRNA expression of CT and DN-EGFR mice under basal conditions or after two weeks of Angiotensin II infusion. Values are expressed relative to those for UBC ± SEM, n = 3–6 mice per condition. *p<0.05, Angiotensin II *versus* corresponding Sham. **C:** Heart weight to tibia length ratio of CT and DN-EGFR mice under basal conditions or after two weeks of Nephrectomy-Aldo-Salt treatment (NAS). Values are means ± SEM, n = 5–6 mice per group. *p<0.05, NAS-treated mice *versus* corresponding Sham. **D:** Cardiac ANF mRNA expression of CT and DN-EGFR mice under basal conditions or after two weeks of NAS treatment. Values are expressed relative to those for UBC ± SEM, n = 5–6 mice per condition. *p<0.05, NAS *versus* corresponding Sham.

DN-EGFR mice constitutively express a cardiomyocyte-specific dominant-negative form of EGFR. As previously reported [9] and confirmed in the present study (Fig. 1; Table 1), the constitutive DN-EGFR expression did not modify cardiac function, morphology or size. In CT mice, two weeks of AngII perfusion induced a cardiac hypertrophy as revealed by the increase (+13%, *p*<0.05) of heart weight/tibia length (HW/TL) ratio, but failed to induce the same phenotype in DN-EGFR mice (Fig. 1a). In the same way, cardiac gene expression of Atrial Natriuretic factor (ANF), a marker of cardiomyocyte stretch, was also increased (+87%, *p*<0.05) in CT but not in DN-EGFR mice (Fig. 1b). These data confirm that EGFR activation is required for AngII induced cardiac remodelling.

### DN-EGFR mice and NAS treatment

The nephrectomy/aldosterone/salt treatment (thereafter called NAS) induces hypertension and cardiovascular remodelling [11] and was used to evaluate the response of mice with defective EGFR signalling to chronic aldosterone challenge. Two weeks of NAS led to renal hypertrophy (Table 2) but did not alter HW/TL ratio of both CT and DN-EGFR mice (Fig. 1c). The left ventricle (LV) end-diastolic diameter/ body weight ratio estimated by echocardiography indicated the absence of LV dilation (Table 3). Echocardiographic assessment of systolic function with several parameters including ejection fraction and tissue Doppler imaging revealed no systolic dysfunction in NAS treated animals. In contrast, we detected a mild alteration of diastolic function in NAS groups as indicated by the increased isovolumic relaxation time (IVRT) but no significant alteration of tissue Doppler parameters (Ea, Epw) (Table 3). Although cardiac hypertrophy was still not developed the expression of ANF was increased similarly by NAS treatment in both CT and DN-EGFR mice (Fig. 1d).

**Table 2:** General characteristics of CT and DN-EGFR mice after 2 or 4 weeks of aldosterone (NAS) treatment.

	CT sham	CT NAS	DN-EGFR sham	DN-EGFR NAS
*2 weeks of treatment*				
n	6	5	5	4
BW (g)	25±1.4	25±1.1	26±0.4	25±0.7
TL (mm)	16.3±0.1	16.4±0.2	16.5±0.2	16.5±0.1
KW/TL (mg/mm)	14.2±0.8	13.7±0.3	16.9±0.5*	16.8±0.8*
*4 weeks of treatment*				
n	5	4	5	4
BW (g)	33±0.3	34±0.2	34±1	32±1
TL (mm)	17.3±0.4	17.1±0.2	17.0±0.3	16.8±0.4
KW/TL (mg/mm)	21.7±0.7	21.9±0.3	29.9±1.5*	27.9±1.9*
SBP (mmHg)	111±2	112±2	134±3*	139±4*

BW: body weight; TL: tibia length; KW: kidney weight; SBP: Systolic Blood Pressure.

*p<0.05 *vs* corresponding Sham.

**Table 3:** Echocardiographic data of DN-EGFR or CT mice after 2 weeks of aldosterone treatment (NAS).

	CT sham	CT NAS	DN-EGFR sham	DN-EGFR NAS
n	6	5	5	5
Body Weight (g)	24±0.8	24.1±0.5	23.8±0.9	24±0.6
Age (day)	68±4	67±3	65±2	71±3
Heart rate (bpm)	506±12	474±9	537±15	470±25
*Cardiac and vascular remodelling*				
Diastolic Ao (mm)	1.36±0.04	1.25±0.05	1.3±0.08	1.32±0.05
LA (mm)	2.47±0.05	2.51±0.12	2.37±0.1	2.7±0.11
LV EDD/BW (mm/g)	0.16±0.01	0.15±0.005	0.16±0.01	0.15±0.003
LV mass/BW (mg/g)	4.14±0.35	4.49±0.2	5.0±0.23	4.17±0.20
*LV Systolic function*				
EF (%)	90±2	87±3	83±3	85±2
Vcfc (circ/s)	3.46±0.17	2.99±0.19	2.9±0.25	2.66±0.11
Sa (cm/s)	2.52±0.28	2.74±0.08	2.51±0.09	2.34±0.16
Spw (cm/s)	3.06±0.16	2.48±0.12	3.08±0.17	2.41±0.14*
*LV Diastolic function*				
IVRT (ms)	16±0.56	20.4±1.54*	17±1.2	19±1.04*
Ea (cm/s)	4.6±0.45	4.59±0.21	4.63±0.12	4.04±0.23
Epw (cm/s)	4.27±0.25	4.78±0.27	5.1±0.29	4.44±0.32
E/Ea	22.8±1.52	21.8±0.64	22.1±1.25	26.5±2.2

LA: left atrium; LV EDD: left ventricle end diastolic diameter; BW: body weight; EF: ejection fraction; Vcfc: velocity shortening of circumferential fibers; Sa, Spw: maximal systolic velocity of the mitral annulus and posterior wall; IVRT: isovolumic relaxation time; Ea and Epw: maximal diastolic velocity of the mitral annulus and the posterior wall; E: maximal velocity of the LV inflow.

*p<0.05 *versus* CT-Sham.

These data suggest that when EGFR activation is defective in cardiomyocytes, it does not affect cardiac remodelling in response to aldosterone challenge. However, although the duration of our treatment was similar to that of AngII treatment, it was too short to induce cardiac hypertrophy in CT mice. We thus extended treatment duration (two more weeks) to induce cardiac hypertrophy in CT mice and get a more robust conclusion about the role of EGFR in cardiac effects of aldosterone.

Under basal conditions, blood pressure was similar between CT and DN-EGFR mice and similarly affected by NAS: systolic blood pressure increased with the same amplitude in both genotype (CT: +21%, DN-EGFR: +24%, *p*<0.05 *vs* respective sham) (Table 2). Renal hypertrophy was also induced to a similar extent in CT and DN-EGFR mice (Table 2).

**Figure 2:**
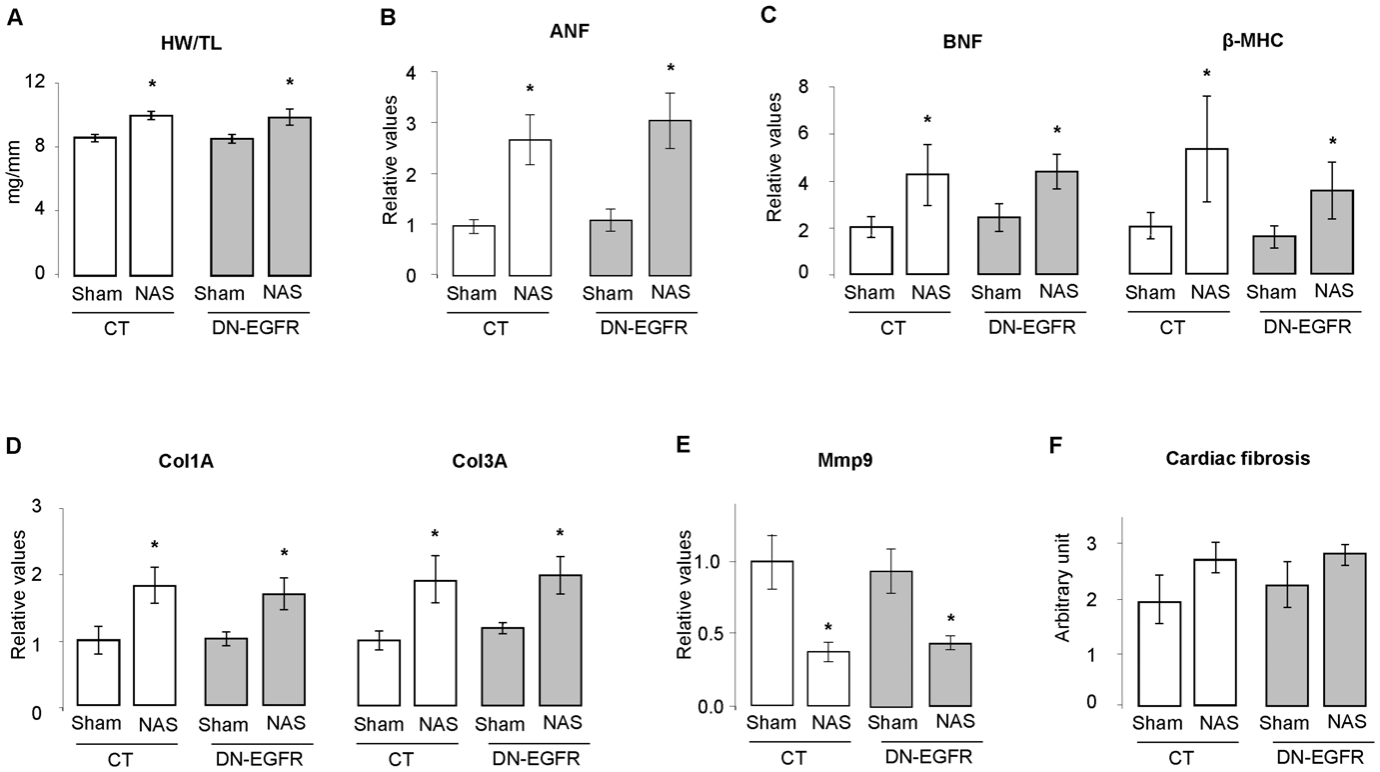
Cardiac phenotype of CT and DN-EGFR mice after 4 weeks of aldosterone (NAS) treatment. **A**- Heart weight to tibia length ratio of CT and DN-EGFR mice under basal conditions or after 4 weeks of NAS treatment. Values are means ± SEM, n = 3–5 mice per group. *p<0.05, NAS *versus* corresponding Sham. **B:** Cardiac ANF mRNA expression of CT and DN-EGFR mice under basal conditions or after 4 weeks of NAS. Values are expressed relative to those for UBC ± SEM, n = 3–5 mice per condition. *p<0.05, NAS *versus* corresponding Sham. **C–E:** Cardiac BNF and βMHC (C) , Col1A and Col3A (D) and Mmp9 (E) mRNA expression of CT and DN-EGFR mice under basal conditions or after 4 weeks of NAS. Values are expressed relative to those for UBC ± SEM, n = 3–5 mice per condition. *p<0.05, NAS *versus* corresponding Sham. **F:** Interstitial cardiac fibrosis quantification. Cardiac fibrosis in CT and DN-EGFR mice under basal conditions or after 4 weeks of NAS. Values are expressed as arbitrary units. n = 3–5 mice per condition.

As expected, one month of NAS led to cardiac hypertrophy in CT mice (Fig. 2a) but also in DN-EGFR mice (CT: +17%, DN-EGFR: +16%, *p*<0.05 *vs* respective sham). Mineralocorticoid receptor expression (mRNA) was not altered (data not shown). Echocardiographic parameters were similar to those obtained by the shorter treatment: NAS treatment altered midly diastolic but no systolic function in both CT and DN-EGFR mice (Table 4). As for shorter treatment, NAS similarly increased cardiac ANF, BNF and β-MHC expression in CT and DN-EGFR mice (Fig 2.b–c). The cardiac expression of collagen 1A and 3A isoforms known to be increased by NAS was similar at basal state between CT and DN-EGFR mice and similarly increased in both groups after one month of NAS (Fig. 2d), whereas the expression of matrix metallopeptidase 9 (Mmp9) a matrix degrading enzyme was decreased by NAS treatment, similarly between control and DN-EGFR mice (Fig. 2e). A discrete, but not significant, increase in cardiac fibrosis was observed in both groups after one month NAS (Fig. 2f). All together, these data indicate that when EGFR activation is defective in cardiomyocytes, it does not affect the cardiac molecular remodelling in response to aldosterone challenge, while it does in response to AngII. Taken together these observations do not support the hypothesis that cardiac EGFR signalling plays a role in aldosterone-induced cardiac pathology.

**Table 4:** Echocardiographic data of DN-EGFR or CT mice after 4 weeks of Nephrectomy-Aldo-Salt treatment (NAS).

	CT sham	CT NAS	DN-EGFR sham	DN-EGFR NAS
n	5	5	4	4
Body Weight (g)	35±0.16	34.6±0.65	34.7±0.38	32.8±0.5
Age (day)	133±0.66	135±1.3	129±0.1	135±1.5
Heart rate (bpm)	572±8.9	515±17*	529±9.8	550±6
*Cardiac and vascular remodelling*				
Diastolic Ao (mm)	1.37±0.05	1.44±0.03	1.42±0.07	1.4±0.09
LA (mm)	2.39±0.10	2.3±0.11	2.52±0.4	2.44±0.09
LV EDD/BW (mm/g)	0.12±0.002	0.12±0.002	0.13±0.002	0.13±0.004
LV mass/BW (mg/g)	4.61±0.23	4.6±0.36	5.39±0.46	4.96±0.39
*LV Systolic function*				
EF (%)	79±2	82±2	82±2	74±3
Vcfc (circ/s)	2.81±0.13	2.92±0.22	3.11±0.017	2.6±0.19
Sa (cm/s)	3.05±0.05	2.76±0.3	2.67±0.17	2.68±0.27
Spw (cm/s)	2.93±0.12	3.02±0.22	2.93±0.17	2.76±0.29
*LV Diastolic function*				
IVRT (ms)	14±0.57	17.2±0.91*	16±0.01	18±0.71*
Ea (cm/s)	5.34±0.33	5.29±0.42	4.67±0.11	4.63±0.3
Epw (cm/s)	5.21±0.18	5.66±0.16	4.6±0.25	5.71±0.29
E/Ea	20.4±1.22	19.5±1.23	21.5±0.36	23.9±1.12

LA: left atrium; LV EDD: left ventricle end diastolic diameter; BW: body weight; EF: ejection fraction; Vcfc: velocity shortening of circumferential fibers; Sa, Spw: maximal systolic velocity of the mitral annulus and posterior wall; IVRT: isovolumic relaxation time; Ea and Epw: maximal diastolic velocity of the mitral annulus and the posterior wall; E: maximal velocity of the LV inflow.

*p<0.05 *versus* CT-Sham.

## Discussion

The molecular and cellular mechanisms underlying the deleterious effects of aldosterone in heart are not fully understood. Interactions between aldosterone and the activation of the Epidermal Growth Factor Receptor (EGFR) is an attractive hypothesis which was essentially based on experiments performed on cultured cells [6]. However, this relation appeared recently to play an important role *in vivo* in the functional effects of aldosterone on vasculature [8]. The aim of our study was therefore to evaluate this possible interaction in the heart *in vivo*, using a transgenic model of EGFR-deficient activation.

Analysing the role of EGFR *in vivo* is not easy since this receptor is involved in many cellular processes. Total EGFR inactivation using global targeted gene inactivation leads to developmental abnormalities or lethality depending of the genetic background of the animal [12], and its chronic pharmacologic inhibition using tyrosine kinases inhibitors induces serious side effects such as diarrhoea, weight loss and death (personal data and [13]). To avoid these effects, we used a mouse model with cardiomyocyte-targeted expression of a dominant-negative EGFR (DN-EGFR). Furthermore, the use of this model allowed discriminating between EGFR inactivation effects restricted to cardiomyocytes or in all cardiac cell types.

EGFR specific tyrosine kinases inhibitors like gefitinib and erlotinib received regulatory approval for use in cancer patients [14]. However although the long-term physiological consequences of suppressed EGFR activity are unknown, cardiac toxicity is already a concern [15]. We previously reported that EGFR deficiency in all cardiac cell types (*waved-2* mice) led to mild cardiac hypertrophy [8] whereas the cardiomyocyte-specific decrease in EGFR activity (DN-EGFR mice) did not. This suggests that non-cardiomyocyte cells could be at the origin of cardiac side effects related to EGFR inhibition in human therapeutics.

Recent *in vivo* and *in vitro* studies provide strong evidence of an interaction between MR and the EGFR signalling pathways, showing both induction of transactivation and expression of the EGFR by aldosterone. Evidence comes mainly from pharmacological experiments showing that different inhibitors of the EGFR tyrosine kinase abolish aldosterone-induced ERK1/2 phosphorylation, and also further downstream events like sodium proton exchanger activation, DNA synthesis or ACE gene expression in MDCK cells, CHO cells, HEK cells, VSMC, and endothelial cells [16] [17] [18] [19] [20] [21]. In addition to aldosterone-induced EGFR transactivation in cell culture, a MR-dependent increase of EGFR mRNA or protein has been reported. *In vitro*, aldosterone enhanced EGFR expression in cultured kidney and human vascular smooth muscle cells [22]–[23]; and *in vivo* EGFR expression is enhanced by aldosterone in the aorta, heart and kidney but not in liver or adipose tissue of adrenalectomized rats. These data suggested an important functional interaction between aldosterone and EGFR-mediated pathways. In this study, we report that EGFR is not required for aldosterone/salt-induced cardiac hypertrophy and molecular remodelling. In the same way, we previously reported that EGFR did not affect the vascular hypertrophy and molecular remodelling induced by aldosterone/salt although it mediates the vascular dysfunction induced in this model [8]. Therefore the remodelling effects of aldosterone do not require EGFR. To date, the interactions between aldosterone/salt and EGFR *in vivo* seem thus restricted to vascular functional effects. Most studies were conducted with vascular cells (endothelial and smooth muscle) [19] [20] [21]; further studies using cardiomyocytes could help to decipher the interactions between EGFR and MR at the cardiac level.

Cardiac fibrosis is a hallmark of the NAS model. After one month NAS, we did not find a significant increase of fibrosis, despite a trend. Indeed, cardiac fibrosis is a dynamic process, which requires time to develop. Other studies report no increase in cardiac fibrosis following 4 weeks of NAS treatment [24] [25]. However, the altered expression of genes of extracellular matrix composition and homeostasis (increased expression of collagens and decreased expression of Mmp9) suggests that the process of cardiac remodelling was initiated, but not affected by the altered EGFR signalling.

We recently reported evidence that interactions between systemic AngII and cardiac MR-mediated pathways lead to increased fibrosis and cardiac remodelling [10], possibly as a consequence of a cumulative action on oxidative stress in the absence of inflammation. Combined increases of systemic AngII and cardiac MR signalling were associated with LV hypertrophy and diastolic dysfunction, and this was prevented by pharmacological MR antagonism [10]. Furthermore, there is increasing evidence that AngII, acting via AT_1_R, may mediate or even exacerbate the damaging effects of aldosterone as evidenced by the beneficial effects of pharmacological AT_1_R blockade on aldosterone induced remodelling [26], [27]. Aldosterone mediated damages, at least in part, seem thus related to AT_1_R activated pathways. However, our results suggest that AngII and NAS induce cardiac hypertrophy via distinct pathways. Indeed, EGFR transactivation is a key process in the AngII-mediated cardiac remodelling as AngII is unable to induce cardiac hypertrophy when cardiomyocyte EGFR activation is defective [9]. In contrast, NAS induce cardiac hypertrophy even when EGFR activation is defective in cardiomyocytes. This result is in accord with a previous study where AT_1_R blockade has no effect on NAS induced cardiac hypertrophy [26]. AngII-induced cardiac hypertrophy is thus EGFR-dependent whereas that induced by aldosterone/salt is EGFR-independent.

We demonstrate in the present study that EGFR activation does not play a significant role in the chronic pathophysiological consequences of aldosterone in the heart, at least in the NAS protocol. Our experimental approach does not exclude a role of EGFR in the rapid, nongenomic effects of aldosterone or a role of EGFR transactivation by aldosterone *in vivo* in other cell types, in particular in smooth muscle cells. Further studies will address this point.
